# Supplementary material for: Incentives behind and Experiences of Being Active in Working Life after Age 65 in Sweden
Source: Int J Environ Res Public Health. 2022 Nov 22;19(23):15490. doi: 10.3390/ijerph192315490 (PMC9740199; doi:10.3390/ijerph192315490)
Supplement: Supplementary file 1 [file ijerph-19-15490-s001.zip › Supplementary File S1_ Interviewguide.pdf]

**INTERVIEW GUIDE**

**Background data**

Sex____________________________

Age___________________________

Residence________________________(rural/urban)

Marital status/Living situation ______________________________

Education_______________________

Occupation________________________ (profession/workplace during working life)

Current employment ______________________________(if working, to what extent)

Perceived health status, any illnesses/diseases ___________________________________

**Individual interview**

Think back in time, when you turned 65. Can you tell me how you made your decision to continue working?

What influenced your decision?

Examples of follow-up questions:

How did your social situation influence your possibilities/willingness to continue working?

How did the work environment influence your possibilities/willingness to continue working?

How was your decision to continue working influenced by your perceived health?

What factors have enabled and facilitated you to continue working after you turned 65?

Tell me how you experience/experienced your working life right now/during the last time you worked? Tell me about a typical working day.

What do you consider is a health-promoting working life for you? What in your working life can promote your health/well-being?

Could you describe if and how your work and your work environment affects your health?

*Possible follow-up questions: Can you give any examples of that? Could you elaborate more on that?*
