# Supplementary material for: Incentives behind and Experiences of Being Active in Working Life after Age 65 in Sweden
Source: Int J Environ Res Public Health. 2022 Nov 22;19(23):15490. doi: 10.3390/ijerph192315490 (PMC9740199; doi:10.3390/ijerph192315490)
Supplement: Supplementary file 1 [file ijerph-19-15490-s001.zip › Supplementary file S2_Overview of the analysis process .pdf]

Supplementary File S2: Overview of the analysis process

**Condensed Meaning Unit**	**Code**	**Category**	**Subtheme**	**Theme**
It has given me so much more than I thought, with the colleagues, a fellowship	Fellowship	Socially enriching	Sustained external resources	Working increases feelings of vitality
I wanted to continue working, I enjoy the social contacts, my husband is gone/dead, why would I sit at home?	A social context			
I get to meet younger people through my work, if I were retired, I would mostly meet people my own age.	Meet younger people			
Because of my job, I have better daily routines, it's the same when it comes to shopping, cooking, and things like that.	Routines	Maintained good everyday routines		
I manage well on my pension, with rent and bills, but I also want to travel, And continue as I lived before. To do that little extra, which a person needs for life to be as good as possible.	Extended finances	Beneficial to my economy		
I feel alert and have heard of those who have withdrawn and retired, then they have deteriorated. It is important to keep going, to use the mind and yes, to keep going.	Use the mind	Good for my mind/cognitive function	Sustained internal resources	
I'm glad my body can handle it, but I also think it's a strength to keep on, not just sit down, for me it's uplifting.	Physically uplifting	Good for my physical ability		
I have more energy and can do much more at home and I feel happier.	Energizing	Increases my vigor		
I was going to stop working when I turned 65, but then they started calling and asking if I could do a little bit here and there, and it was fun.	Required	I am still "needed"	Adds meaningfulness	
And then this... it's the creativity of sorts, that is incredibly important to me. We (hairdressers) are artists, that's how it is.	Creative work	Work satisfaction		
It feels good, I thought it would be hard and heavy work, but I think there's more complaining from my friends who are 20 years younger, the others say I have the capacity of two 30-year-olds.	Capacity	I am still capable		
I haven't taken much of a burdensome job. Not been to conferences or had that much parental contact, only been with the children, which I like the most.	Choose tasks	I decide for myself what	Having flexible working conditions	
It's wonderful because I get to decide for myself when I work. I can say no because I must do something else, I don't have time, then I say no.	Can say no	I decide for myself when		
Maybe I will work 50% or 75%, but I get to decide for myself, and it is beneficial for me.	Choose extent	I decide for myself how much		
